# Molecular Targeting of the Fibroblast Growth Factor Receptor Pathway across Various Cancers

**DOI:** 10.3390/ijms25020849

**Published:** 2024-01-10

**Authors:** Khine S. Shan, Shivani Dalal, Nyein Nyein Thaw Dar, Omani McLish, Matthew Salzberg, Brian A. Pico

**Affiliations:** Memorial Health Care, Division of Hematology and Oncology, Pembroke Pines, FL 33028, USA; sdalal@mhs.net (S.D.); nthawdar@mhs.net (N.N.T.D.); ommclish@mhs.net (O.M.); msalzberg@mhs.net (M.S.);

**Keywords:** FGF, FGFR, fibroblast growth factor receptor, fibroblast growth factor, targeted therapy, genomic profiling, personalized medicine

## Abstract

Fibroblast growth factor receptors (FGFRs) are a family of receptor tyrosine kinases that are involved in the regulation of cell proliferation, survival, and development. FGFR alterations including amplifications, fusions, rearrangements, and mutations can result in the downstream activation of tyrosine kinases, leading to tumor development. Targeting these FGFR alterations has shown to be effective in treating cholangiocarcinoma, urothelial carcinoma, and myeloid/lymphoid neoplasms, and there are currently four FGFR inhibitors approved by the Food and Drug Administration (FDA). There have been developments in multiple agents targeting the FGFR pathway, including selective FGFR inhibitors, ligand traps, monoclonal antibodies, and antibody–drug conjugates. However, most of these agents have variable and low responses, with some intolerable toxicities and acquired resistances. This review will summarize previous clinical experiences and current developments in agents targeting the FGFR pathway, and will also discuss future directions for FGFR-targeting agents.

## 1. Introduction

Fibroblast growth factor (FGF) receptors (FGFRs) are a family of five receptor tyrosine kinases (RTKs) named FGFR1-5. FGFRs have an extracellular domain that binds to FGF ligands, transmembrane, and intracellular tyrosine kinase domains, except for FGFR5, which lacks an intracellular tyrosine kinase (TK) domain [1,2,3]. The activation of FGFRs by FGFs plays an essential role in cellular proliferation, migration, survival, embryonic development, metabolism, homeostasis, tissue repair, and apoptosis maintenance [4]. Dysregulation in the FGFR signaling pathways caused by *FGFR* gene amplifications, mutations, and fusions leads to oncogenesis, tumor progression, and angiogenesis in the tumor microenvironment (TME), as well as resistance to anticancer treatment [1,3,4]. According to a report of the next-generation sequencing (NGS) study of 4853 solid tumors, approximately 7.1% of cancers are caused by FGFR aberrations, including 66% by gene amplifications, 26% by mutations, and 8% by rearrangements [5]. As we have a greater understanding of the role of FGFR and its inhibitors in oncogenesis, there are gradual developments in FGFR-targeting therapies in various types of cancers. FGFR inhibitors can be divided into small-molecule oral tyrosine kinase inhibitors (TKIs), ligand traps, monoclonal antibodies, and antibody–drug conjugates (ADCs) [6,7,8]. Currently, there are four FDA-approved FGFR inhibitors used to treat cholangiocarcinoma (CCA), urothelial tumors, and myeloid/lymphoid neoplasms (MLNs). This review will discuss the FGFR signaling pathway, *FGFR* alterations across various types of cancer, current FDA-approved FGFR inhibitors, other selective FGFR and non-selective FGFR multi-tyrosine kinase inhibitors (multi-TKIs), previous and current developments in FGFR ligand traps, monoclonal antibodies, and antibody–drug conjugates (ADCs), and their combination treatments, resistance mechanisms, challenging issues, and future directions.

## 2. FGF/FGFR Signaling Pathway

FGFs and their signaling pathway participate in a wide variety of cellular processes including cell proliferation, differentiation, survival, migration, tissue remodeling, and angiogenesis. *FGFR* gene amplification, overexpression, point mutations, and chromosomal translocations can promote cancer development and progression [9]. In murine and mammalian genomes, 22 FGF ligands have been identified. Eighteen mammalian ligands have been identified, and they are divided into six subfamilies based on their phylogeny and sequence homology [9,10]. The subfamilies include five paracrine subfamilies, FGF1 (FGF1 and FGF2), FGF4 (FGF4, FGF5, and FGF6), FGF7 (FGF3, FGF7, FGF10, and FGF22), FGF8 (FGF8, FGF17, and FGF18), and FGF9 (FGF9, FGF16, and FGF20), and one endocrine subfamily, FGF19 (FGF19, FGF21, and FGF23) [10]. FGFs show a high binding affinity for heparin and FGFRs. The binding of FGFs to the inactive monomeric FGFRs triggers conformational changes in the receptors, resulting in the dimerization and activation of the cytosolic tyrosine kinases by phosphorylating the tyrosine residues within the cytosolic tail of the FGFRs. Canonical FGFs are tightly bound to heparin/heparin sulfate (HS) proteoglycans (HSPGs), and they function to limit diffusion through the extracellular matrix (ECM) and serve as cofactors that regulate affinity for FGFR signaling [9].

**Figure 1:**
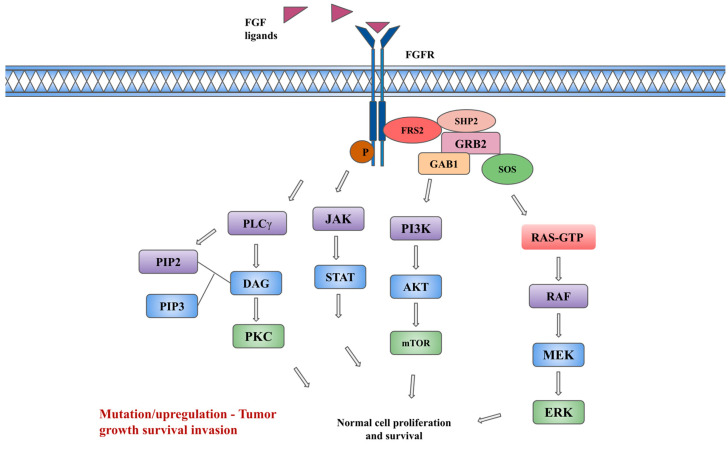
FGF/FGFR and downstream signaling pathway. Description: The binding of FGF ligands to FGFRs leads to receptor dimerization, conformational changes, and phosphorylation (P in figure) of kinases, leading to the interaction of adaptor protein FRS2 (FGFR substrate 2) with SHP2 (Src homology-2 domain-containing protein tyrosine phosphatase-2), GRB2 (growth factor receptor-bound 2), SOS (son of sevenless). This subsequently leads to the downstream activation of the PI3K, and MAPK signaling pathways. In addition, the activation of JAK-STAT and protein kinase C (PKC) pathways occurs independently of FRS2. The activation of the PKC pathway occurs via binding of phospholipase C-gamma (PLCγ) to phosphotyrosine, causing hydrolyzation of PIP2 (Phosphatidylinositol 4,5-bisphosphate) to PIP3 (Phosphatidylinositol (3,4,5)-trisphosphate) and DAG (diacylglycerol), which in turn leads to activation of PKC and MAPK pathways.

The activation of FGFRs by FGFs causes the downstream activation of RAS/RAF/MEK/ERK kinases in the MAPK (mitogen-activating protein kinase) pathway, PI3K-AKT-mTOR kinases in the PI3K (phosphoinositide 3 kinase) pathway, and the JAK-STAT (Janus kinase/signal transducer and activator of transcription) pathway, and protein kinase C (PKC) via phospholipase C-gamma (PLCγ) [1,7]. Regulation mechanisms to attenuate the aforementioned signaling pathways are available. The SPRY (Sprouty) family of proteins, which are MAPK phosphatases, directly binds to RAF and causes the inhibition of subsequent MAPK signaling. Notably, FGF signaling activates these SPRY proteins, which may serve as a form of auto-inhibition. A non-tyrosine kinase FGFR (FGFRL1), which can bind FGF ligands and possibly function as a decoy receptor or modulator of receptor turnover or signaling, represents another negative regulation pathway [9,11]. Downstream of the signaling tyrosine kinase FGFRs, intracellular signaling cascades are also tightly regulated by specialized adaptor proteins such as FGFR substrate 2α (FRS2α). Each pathway regulates specific cellular behaviors such as cellular proliferation, embryonic development, endocrine homeostasis, and tissue repair. The inappropriate expression of FGF and the activation of FGFRs are associated with various pathologic conditions, unregulated cell growth, and tumorigenesis [12]. Figure 1 depicts the FGF/FGFR signaling pathway.

## 3. FGFR Signaling Diversity in Cancer

The FGFR pathway is an oncogenic signaling pathway and its dysregulation is associated with the pathogenesis of various malignancies. FGFR alterations in cancers can be broadly classified into three main types: FGFR gene amplification, gene mutation, and gene fusion. *FGFR1* amplification is the most common *FGFR* alteration (42%) [5]. Uehara et al. reported that patients who harbor *FGFR* alterations have worse overall survival (OS) than those without and 94% of those who harbor *FGFR* alterations also have other genomic co-alterations, including the TP53 axis, the PI3K pathways, and the MAPK pathways [2]. FGFR inhibition has been noted to inhibit cell proliferation and cause cell death in a multitude of in vitro and in vivo tumor models harboring *FGFR* alterations; thus, FGFR has been the target for cancer drug development [9].

### 3.1. Gynecologic Cancers (Cervical, Ovarian, and Endometrial)

*FGF1* amplification has been shown to promote angiogenesis and reduce disease-free progression as well as OS in patients with ovarian cancer [13,14]. It reduces the transcriptional activity of p53 and increases the expression of p21, which subsequently leads to the antiapoptotic activity in response to the treatment for ovarian cancers, thereby leading to the chemotherapy resistance [14]. Individual FGFRs including FGFR2 IIIb are overexpressed in ovarian cancer [15]. Upregulated FGFR2 expression and its association with the transformation of ovarian endometrioma to clear cell carcinoma of the ovary were shown in a study by Taniguchi et al. [16]. Several cases of serous ovarian carcinoma are noted to have high levels of FGFR4 proteins and are interestingly associated with poor survival [17].

FGFR2 expression has been reported to cause cell proliferation and the progression of cervical dysplasia to malignancy. In a study by Choi et al., the immunohistochemical expressions of FGFR1, FGFR2, FGFR3, and FGFR4 in 336 patients with cervical cancer were evaluated and it was confirmed that FGFR2, FGFR3, and FGFR4 expressions were important prognostic indicators in cervical cancer [18].

*FGFR2* alteration is the most common form of FGFR alterations noted in endometrial cancers, thereby making it an attractive therapeutic molecular target [5]. Inhibition of FGFR kinase activity inhibits cell cycle progression, cell survival, and colony formation, leading to cell death [5].

### 3.2. Gastrointestinal Cancers

#### 3.2.1. Cholangiocarcinoma (CCA)

*FGFR2* fusions in CCA were first identified in 2013 by Wu and colleagues [19]. Subsequent studies demonstrated that *FGFR2* fusions occur nearly exclusively in intrahepatic CCA (iCCA) compared to other biliary tract cancers. *FGFR2* fusion frequency in iCCA is approximately 10–15% across multiple tumor genotyping studies [20,21]. The evolution of non-liver fluke-associated CCA is suggested to be associated more with *FGFR2* fusions than in liver fluke-associated CCA as *FGFR* fusions in fluke-associated and non-fluke-associated CCA were 0.8% and 11.6%, respectively (*p* = 0.0006) [22]. Patients with iCCA and *FGFR2* fusions were noted to have a better prognosis and younger age at diagnosis [21].

#### 3.2.2. Gastric and Gastroesophageal Junction Cancers

Helsten et al. performed a comprehensive review of *FGFR* alterations in gastric cancer and noted that *FGFR1* mutations, *FGFR2* amplifications, and *FGFR3* rearrangements are the most common *FGFR* alterations in gastric cancer and they may sometimes be discovered as co-occurring mutations [23]. In a study conducted in China, 5557 Chinese patients with solid organ malignancies were evaluated for the presence of *FGFR1-4* alterations via NGS, which included 254 cases of gastric cancers [24]. The study noted that *FGFR1-4* aberrations occurred in 12.2% of the gastric cancer samples with amplifications being the most frequent alteration, followed by rearrangements and mutations [24]. The most common alterations were detected in the *FGFR2* gene, followed by the *FGFR1* gene, and to a lesser extent in the *FGFR3* and *FGFR4* genes [24]. In another study, 20% (5/25) of gastric cancer was found to carry the potentially targetable *FGFR3-TACC3* (transforming acidic coiled-coil-containing protein 3) fusion [25]. In *FGFR3–TACC3* fusion, the FGFR tyrosine kinase domain is fused to the TACC coiled-coil domain, resulting in constitutive activation of the fused receptor [26]. *FGFR2* gene amplification is the most common aberration (2–9%), which leads to FGFR2 protein overexpression and FGFR pathway constitutive activation in gastric cancer [25]. In patients with early-stage gastric cancer, *FGFR2* amplifications were noted to be associated with a higher-grade tumor stage, more frequent lymph node involvement, and inferior OS [27,28]. In the metastatic setting, *FGFR2* amplifications are also associated with inferior progression-free survival (PFS) and OS in patients receiving platinum and fluoropyrimidine chemotherapy [28,29].

### 3.3. Urothelial Cancers

In urothelial carcinoma, *FGFR3* alterations have been previously documented in nearly 60% of low-grade noninvasive papillary urothelial carcinoma of the bladder, 35.6% of upper tract high-grade urothelial cancer, and 26.7% of overall urothelial carcinoma [30,31,32]. Base substitutions are the most common *FGFR3* alterations (84%) seen in patients with urothelial cancer [33]. The activating missense mutations and in-frame *FGFR3-TACC3* fusions are the most common *FGFR3* alterations in advanced bladder cancer [34]. Ligand-independent dimerization between mutant receptors occurs by gain-of-function missense mutations in the extracellular and transmembrane domains of FGFR3. FGFR3 tyrosine kinase activity is promoted by mutations in the intracellular kinase domain [30,35]. Higher FGFR3 mRNA and protein expression in bladder cancer are associated with missense mutations of FGFR3 [36].

### 3.4. Non-Small Cell Lung Cancer

In a study by Zhou et al., a total of 10,966 patients with non-small cell lung cancer (NSCLC) received NGS of tumor specimens or cell-free tumor DNA [37]. *FGFR* aberrations, including fusions, mutations, and gene amplifications, were detected in 1.9% (210/10,966) of the population, with more prevalence in squamous cell carcinoma of the lung compared to lung adenocarcinoma [37]. The majority of the patients who carried *FGFR* activating and transforming mutations had concurrent mutations in the PI3K pathway genes, including *PIK3CA* and *PIK3R2*. This highlights an intriguing molecular feature and potential development of combination therapies targeting both FGFR and PI3K pathways in patients with *FGFR-altered* NSCLC exhibiting activated PI3K pathways. A total of 24 patients were found to have *FGFR* amplification with *FGFR1* amplification being the most common alteration [37]. It is worth noting that half of the patients with *FGFR* fusions also carried epidermal growth factor receptor (EGFR) aberrations [37]. *FGFR* fusions may act as a mechanism of acquired resistance to EGFR inhibitors in patients who were previously treated with EGFR TKIs [38]. This suggests that concurrent FGFR and EGFR inhibition could overcome the acquired resistance of EGFR inhibitors. A low frequency of FGF19 amplifications was also noted. As *FGF19* encodes the ligand for FGFR4, *FGF19* amplifications correspond with constitutive activation of FGFR4-dependent signaling, which can act as an oncogenic driver and a potential therapeutic target.

### 3.5. Breast Cancers

The amplification of *FGFR1* represents the most frequent genomic alteration in breast cancer, with *FGFR2-4* gene amplifications being less commonly seen [39]. The genomic analysis of The Cancer Genome Atlas (TCGA) and the Molecular Taxonomy of Breast Cancer International Consortium (METABRIC) databases confirmed that the amplification of *FGFR1* is the most common type of *FGFR* alteration, occurring in nearly 14% of patients with breast cancer [40]. Patients with FGFR1 overexpression were noted to have reduced survival rates compared to the remaining cohort of patients [40]. *FGFR1* amplification is also shown to be responsible for the resistant mechanisms to endocrine therapy in breast cancer via both aberrant ligand-dependent and ligand-independent signaling [41]. *FGFR1* amplification and overexpression also contribute to the resistance of breast cancer cells to the CDK4/6 inhibitors used in combination with endocrine therapy in either in vitro or in vivo patient-derived xenograft models [42]. A recent study also demonstrated that the presence of *FGFR* genetic aberrations could predict the occurrence of brain metastases in patients with breast cancer [43]. On the other hand, *FGFR2-4* amplifications represent approximately 1–2% of all breast cancer cases [5]. *FGFR* gene fusions such as *FGFR3-TACC3* may trigger cancer development, allowing the activation of FGFR3 tyrosine kinase [44]. A higher expression of FGFR3 was noted in patients with tamoxifen-resistant estrogen receptor (ER)-positive breast cancers when compared to tamoxifen-sensitive ER-positive breast tumors. FGFR3 stimulation has been found to trigger resistance to tamoxifen via the activation of the PLCγ signaling cascade [45].

### 3.6. Glioblastoma

Glioblastoma (GBM) is the most common primary brain malignancy in adults, with a median onset of approximately 55–60 years. As per the revised World Health Organization (WHO) Classification of the central nervous system (CNS) tumors, glioblastoma refers to CNS WHO grade 4 isocitrate dehydrogenase (IDH) wild-type tumors and astrocytoma refers to CNS WHO grade 4 IDH mutant tumors. Most patients with glioblastoma are treated with a multidisciplinary approach of surgical resection, postoperative radiation, and adjuvant chemotherapy. Despite maximal treatment, the overall outcomes of patients with glioblastoma remain dismal with OS ranging from 1.5 to 2 years. Yamaguchi et al. demonstrated that expression of FGFR1 increases with the WHO grade in astrocytoma [46]. FGFR1α is the predominant isoform in normal brain and low-grade gliomas, while high-grade gliomas had a higher expression of FGFR1β [46,47]. Loss of the FGFR1α exon increases the receptor–ligand affinity and the sensitivity of tumor cells to FGFs present in their environment, thus contributing to GBM development [48]. FGFR1 expression in malignant glioma has also been associated with increased migration of cancer cells from its site of origin [49]. FGFR1 signaling also promotes radioresistance in glioma cell lines through PLCγ1 and hypoxia-inducible factor 1-alpha (HIF1α) [50]. High expression of the ephrin type-A receptor 4 (*EPHA4*) gene in glioma cells was found to potentiate FGF2–FGFR1 signaling and promote cell growth and migration through the AKT/MAPK and RAC1/CDC42 pathways, respectively [50]. FGFR1 signaling promotes radioresistance in glioma cell lines through PLC1γ (Phospholipase C Gamma 1) and HIF1α pathways [50]. In summary, FGFR1 is a key regulator of tumor growth and invasion. It is a cause of therapy resistance in malignant glioma. While FGFR1 is mainly expressed on neurons, FGFR2 is the primary FGFR on astrocytes [51]. In contrast to FGFR1, FGFR2 expression decreases with the glioma grade. Reduced expression of FGFR2, as well as its IIIb and IIIc isoforms, are associated with a higher tumor grade with poor survival in patients with glioma [52]. Loss of FGFR2 is associated with a loss of chromosome 10q, which represents an unfavorable prognosis [53]. Fusion of the *FGFR3* and *TACC3* gene also generates an oncogenic FGFR3 form in a small subset of patients [26].

### 3.7. Gastrointestinal Stromal Tumors and Other Soft Tissue Sarcomas

#### 3.7.1. Gastrointestinal Stromal Tumors

Gastrointestinal stromal tumors (GISTs) are mesenchymal neoplasms that typically arise in the stomach and small intestine, although they can arise in any portion of the gastrointestinal system. Multimodal approaches including resection and the use of targeted therapy such as imatinib and avapritinib have been employed for the management of these tumors. The most common molecular alterations found in GISTs are the KIT and PDGFRA mutations. In the subset of GIST without any alterations of the KIT/PDGFRA/SDH/RAS pathway, targeted sequencing of so-called quadruple wild-type GIST has shown the presence of activating mutations or gene fusions involving FGFR1. One patient had *FGFR1*–*Hook homolog 3* (*HOOK3*) fusion and two patients carried *FGFR1*–*TACC1* fusion transcripts [54,55]. The FGFR pathway has also been shown to be related to the imatinib resistance. FGF2 is overexpressed in imatinib-resistant GIST cells [56]. The interaction of FGF2 with FGFR1 and FGFR3, respectively, restores MAPK signaling during treatment with imatinib and proto-oncogene c-KIT phosphorylation in imatinib-resistant models [57]. A gain of function mutation in *FGFR2* is also a potential mechanism associated with imatinib resistance [58].

#### 3.7.2. Rhabdomyosarcoma

Rhabdomyosarcomas (RMSs) are malignant soft tissue tumors arising from immature cells, which are precursors of striated skeletal muscle. Using modern combined modality therapies such as chemotherapy, surgery, and radiation, the cure rates of RMS have improved substantially. Taylor et al. described *FGFR4* aberrations in primary human RMS [59]. In RMS cell line models, it was observed that these mutations promoted FGFR4 autophosphorylation, STAT3 phosphorylation, and activation of the cell cycle, thereby activating DNA replication pathways. *FGFR4* mutations increased the proliferation rate of the cells and metastatic potential. High expression of FGFR4 mRNA was also associated with worse survival in a clinical cohort of 146 patients [59]. In alveolar RMS cells, FGFR4 stimulation causes degradation of the pro-apoptotic molecule Bcl-2-like protein 11 (BIM) and upregulation of its antagonist B-cell lymphoma-extra-large (Bcl-XL) proteins [60]. The FGFR pathway has been implicated in the development and progression of several other soft tissue sarcoma (STS) subtypes. A comprehensive analysis by Chudasama et al. ultimately revealed FGFR1 copy number gain and overexpression in leiomyosarcomas (LMSs), undifferentiated pleomorphic sarcomas (UPSs), and de-differentiated liposarcoma (DDLPS) as well as other sarcoma subtypes [61]. In cellular models of various histologies of STS, the MAPK signaling axis was found to be the most critical effector pathway mediating FGFR1 signaling [61]. FGFR1 was also shown to be overexpressed in Ewing’s sarcoma, which is a high-grade mesenchymal malignancy of bone or soft tissue [62].

### 3.8. Head and Neck Cancers

*FGFR* aberrations are one of the most frequently occurring RTK genomic alterations seen in head and neck squamous cell carcinoma (HNSCC), making the FGF/FGFR axis a promising target for the development of new treatment options for patients with HNSCC [63]. *FGF/FGFR* genomic alterations can be divided into ligand-dependent aberrations (*FGF* genomic alterations) and ligand-independent aberrations (*FGFR* aberrations). *FGF/FGFR* gene deregulation has been detected in approximately 30–50% of HNSCC [64,65]. Among them, *FGFR1* gene amplification, *FGF3/4/19* gene amplifications, and *FGFR3* mutations are the most frequent *FGF/FGFR* genomic alterations [63]. FGF2 has been reported to be highly expressed in up to 60% of HNSCC [66]. Marshall et al. noted that FGF2 was frequently co-expressed with FGFRs in the majority of the HNSCC cell lines they tested, which can form an autocrine loop to drive oncogenesis [67]. *FGFR1* gene amplification is predominantly detected in HPV-negative patients with HNSCC and is more prevalent in laryngeal papillary (LPSCC) and hypopharyngeal squamous cell carcinoma (HPSCC) [68]. *FGFR2* mutations are mainly enriched in HPV-positive patients with HNSCC, unlike *FGFR1* gene amplification [69]. *FGFR3* mutations have been implicated in about 5.8–24% of patients with HNSCC. *FGFR3-TACC3* fusion is also reported in about 2.5–3.7% of patients with HNSCC [70]. FGFR4 is much less studied than other FGFRs in HNSCC even though FGFR4 is highly expressed in 16–39% of patients with HNSCC [71]. FGFR4 overexpression has been reported to be associated with poorer OS of patients with HNSCC [72].

## 4. Generations of FGFR Inhibitors

Given a similar structure to adenosine triphosphate (ATP), oral TKIs compete for the ATP binding cleft of the kinase domain on the FGFR receptor. Reduction in tyrosine kinase phosphorylation by competitive reversible inhibition leads to the blockade of multiple downstream pathways, thus causing inhibition of cell proliferation [6]. There are close similarities in the ATP binding site of intracellular kinase domains among the RTK family. Thus, first-generation FGFR inhibitors are non-selective inhibitors against multiple kinases (PDGFRs, VEGFRs, KIT, and RET), which include ponatinib, lucitanib, dovitinib, lenvatinib [6,7]. Multi-kinase FGFR inhibitors can lead to various adverse effects due to low specificity and multi-target effects, thus leading to the development of more specific and selective TKIs for the FGFR pathway [6].

Second-generation FGFR inhibitors are more selective including FGFR1-3 inhibitors (pemigatinib, infigratinib, AZD4547, Debio1347), FGFR1-4 inhibitors (erdafitinib, rogaratinib), and a selective FGFR 4 inhibitor (fisogatinib) [7]. Despite their potential anti-tumor activity, they are ineffective at overcoming commonly acquired FGFR gatekeeper mutations (*FGFR1 V561M*, *FGFR2 V564F*, *FGFR3 V555M*, FGFR4 *V550M/L*) and other mutations including an *FGFR1 N546K* mutation and *FGFR2 N550H* mutations [4,73,74]. Third-generation FGFR inhibitors such as futibatinib (TAS-120) can covalently bind to a highly conserved cysteine residue (Cys488 in FGFR1c) in FGFR kinase, and cannot be readily replaced by ATP, thus prolonging the duration of the activity, and overcoming secondary FGFR2-resistant mutations in patients with infigratinib or Debio1347 resistance [7].

### FDA-Approved FGFR Inhibitors

**Figure 2:**
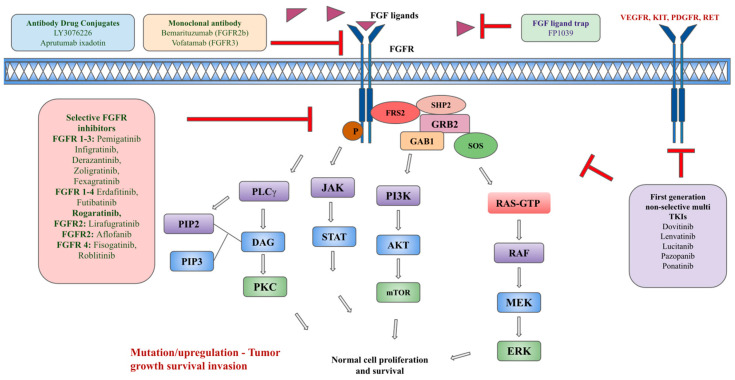
Types of FGFR pathway inhibitors.

The benefit of targeting FGFR has been demonstrated in urothelial cancers, CCA, and MLNs. Currently, there are four FDA-approved FGFR inhibitors but infigratinib has been withdrawn from the market in the United States [75]. Figure 2 summarizes different types of FGFR inhibitors.

*Pemigatinib* is an oral selective reversible ATP competitive FGFR1-3 TKI. FDA approved pemigatinib on 17 April 2020 as a second line for the treatment of adults with previously treated, unresectable locally advanced or metastatic CCA with *FGFR2* fusions or rearrangements [76]. The approval was based on a single-arm phase II FIGHT-202 trial, which evaluated patients with unresectable or metastatic CCA with *FGFR2* gene fusions or rearrangements, who progressed on at least one prior therapy [77]. Patients were divided into three cohorts including cohort 1 with *FGFR2* fusions or rearrangements, cohort 2 with other *FGF/FGFR* alterations, and cohort 3 with no *FGF/FGFR* alterations. The objective response rate (ORR) was 35.5% (95% CI: 26.5–45.4) in cohort 1 patients with *FGFR2* fusions or rearrangements with a duration of response (DOR) of 9.1 months while patients in cohorts 2 and 3 had 0% ORR [77]. Responses were observed only in patients with *FGFR2* fusions or rearrangements and not in other *FGF/FGFR* alterations nor patients without FGFR mutations. The most common adverse effects were hyperphosphatemia, arthralgia, stomatitis, hyponatremia, abdominal pain, fatigue, pyrexia, cholangitis, and pleural effusions, and 4% of patients had serous retinal detachment [77]. The most frequent all-grade and grade 3 or higher adverse events were hyperphosphatemia [77]. Drug discontinuations occurred in 9% and dose reductions occurred in 20% of patients [77]. Updated results from FIGHT-202 presented at ASCO 2021 showed independent, centrally confirmed durable responses, and sustained tolerability, with ORR of 37.0% with median OS of 17.5 months (95% CI: 14.4–22.9) with higher OS in responders (30.1 months vs. 13.7 months) [78]. A phase III FIGHT 302 study is currently being evaluated to use pemigatinib as a first-line treatment in comparison with gemcitabine and cisplatin in patients with unresectable or metastatic CCA and *FGFR2* alterations (fusions/rearrangements) [79,80].

*Pemigatinib* was also investigated in another landmark phase II FIGHT-203 trial in patients with relapsed/refractory (R/R) MLNs with *FGFR1* rearrangements regardless of prior lines of treatment [81]. MLN with *FGFR1* rearrangements could present as a chronic or blast phase with involvement of bone marrow and/or extramedullary disease (EMD) [81]. The most common *FGFR1* fusion partner genes were 13q12/ZMYM2 (45.5%) and 22q11/BCR (24.2%). Complete responses (CRs) were seen in 64.7% of evaluated patients (77.4% per Central Review Committee). The complete cytogenic response was seen in 72.7% of patients with 83.3% CR in chronic-phase patients without EMD and 38.5% in the blast phase with or without EMD [81]. This led to FDA approval of pemigatinib for adults with R/R MLNs with *FGFR1* rearrangements on 26 August 2022 [82]. It is a treatment option for patients with MLN with *FGFR1* rearrangements ineligible for hematopoietic stem-cell transplantation (HSCT) or it may facilitate bridging to HSCT in eligible patients.

*Infigratinib* is another selective oral reversible ATP competitive FGFR1-3 TKI. FDA granted accelerated approval for infigratinib on 28 May 2021, as a second line for adults with previously treated unresectable or metastatic CCA with *FGFR2* fusions or rearrangements [83]. Approval was based on the phase II CBGJ398X2204 trial of patients with advanced or metastatic CCA with *FGFR* genetic alterations who have received at least one prior line of treatment [83,84]. ORR was 23.1% (95% CI: 15.6–32.2) including 1 CR and 24 partial responses (PRs) with a DOR of 5.0 months and median PFS of 7.3 months [85]. ORR was noted to be higher in the second line setting than in the third or later line setting (34% vs. 13.8%) [85]. The most common all-grade adverse events were hyperphosphatemia, eye-related diseases excluding serous retinopathy and retinal detachment, stomatitis, and fatigue. Adverse events are comparable to other FGFR inhibitors. Infigratinib was also compared to gemcitabine plus cisplatin as a front-line setting in patients with advanced or metastatic CCA with *FGFR2* gene fusion/translocation in the phase III PROOF trial (NCT03773302). However, the study was terminated as infigratinib was withdrawn from the market on 31 March 2023, due to difficulties in enrolling participants for the required confirmatory trial and not due to safety or efficacy concerns [86].

*Erdafitinib* is an oral reversible inhibitor of FGFR1-4, which was FDA approved on 12 April 2019 as a second line for patients with locally advanced or metastatic urothelial cancer with *FGFR2/3* alterations who have progressed on prior platinum-containing chemotherapy [87]. The approval was based on the phase II BLC2001 trial in which erdafitinib was evaluated in pretreated patients with unresectable or metastatic urothelial cancers with *FGFR* alterations [88]. Participants had *FGFR3* gene mutations (*G370C, R248C, S249C, Y373C*) or *FGFR* gene fusions (*FGFR2-BICC1, FGFR2-CASP7, FGFR3-TACC3, FGFR3-BAIAP2L1*) [88]. ORR was 40% (95% CI: 31–50) with CR in 3% and PR in 37%. Median PFS was 5.5 months (95% CI: 4.2–6.0) and median OS was 13.8 months (95% CI: 9.8–not reached) [88]. The response rates were 49% among patients with *FGFR* mutations and 16% among those with *FGFR* fusions, and response rates were not affected by a particular *FGFR* mutation [88]. The most common all-grade adverse effects were similar to other FGFR inhibitors including hyperphosphatemia, stomatitis, diarrhea, dry mouth, and dysgeusia [88]. In total, 27% of patients who were treated with erdafidinib had central serous retinopathy but 63% of those patients had resolution at the data-cutoff time. There seems to be less response to immunotherapy in patients with *FGFR* mutations or fusions but 59% had a response to erdafitinib after failure of immunotherapy compared to 40% in patients with no exposure to immunotherapy [88]. Updated results in 2022 showed durable efficacy with 40% ORR at a median follow-up of 24 months and a manageable safety profile [89].

*Erdafitinib* is currently being evaluated in a confirmatory phase III THOR trial evaluating patients with metastatic or unresectable urothelial carcinoma with *FGFR2/3* alterations (mutations/fusions) who progressed after 1–2 prior lines of treatment to obtain full FDA approval and the application is being submitted as of August 2023 [90]. The most common *FGFR* mutations included in the study were *FGFR3 S249C, FGFR3 Y373C, FGFR3 R248C,* and *FGFR3 G370C*, while the most common *FGFR* fusions were *FGFR3-TACC3-V1, FGFR3-TACC3*, and *FGFR3-TACC-V3* [91]. The THOR trial divides patients into two cohorts. Cohort one compares erdafitinib vs. the standard of care (docetaxel or vinflunine) after at least one prior line of treatment including an anti-PD-L1 (programmed death-ligand 1) agent, while cohort two compares erdafitinib vs. pembrolizumab after one prior line not containing a PD-L1 agent [91,92]. In total, 70% of patients had visceral metastases and 90% were low in PD-L1 (combined positive score—CPS < 10). Loriot et al. recently reported an analysis of cohort 1 after a median follow-up of 15.9 months [91,92]. Primary endpoint OS was met with 12.1 months in the erdafitinib group vs. 7.8 months in the chemotherapy group (hazard ratio for death (HR): 0.64; 95% CI: 0.47–0.88; *p* = 0.005) with improved PFS of 5.6 vs. 2.7 months (HR: 0.58; 95% CI: 0.44 to 0.78; *p* < 0.001) and improved ORR of 46% vs. 12% (relative benefit: 3.94; 95% CI: 2.37–6.57) in the chemotherapy group [91,92]. The most common all-grade adverse effects were hyperphosphatemia, diarrhea, stomatitis, dry mouth, and palmer plantar erythrodysesthesia syndrome and 23 patients had central serous retinopathy. More treatment-related adverse effects leading to dose reductions were observed in the erdafitinib group (66% vs. 21%) but more discontinuations were observed in the chemotherapy group (8.1% vs. 13%) and grade 3 or 4 adverse effects of central serous retinopathy were seen in 2.2% of patients in the erdafitinib group [91,92]. However, no significant OS was observed in cohort 2 between erdafitinib and pembrolizumab with median OS of 10.9 months in the erdafitinib group vs. 11.1 months in the pembrolizumab group (HR: 1.18, 95% CI: 0.47–0.88; *p* = 0.18) even though erdafitinib had numerically longer PFS of 4.4 months compared to 2.7 months in the pembrolizumab group (HR: 0.88, 95% CI: 0.70–1.10) and higher ORR of 40.0% compared to 21.6% with the pembrolizumab group (95% CI: 1.32–2.39; *p* < 0.001) [93].

*Futibatinib* (TAS-120) is a next-generation, highly selective irreversible TKI that covalently binds FGFR1-4, and it has been shown to overcome the acquired resistant mutations observed at progression with other FGFR inhibitors including pemigatinib and infigratinib [94]. Futibatinib had accelerated FDA approval on 30 September 2022 for previously treated adult patients with unresectable, locally advanced, or metastatic intrahepatic CCA with *FGFR2* fusions or rearrangements. It is the third FGFR inhibitor to receive approval for patients with CCA who harbor *FGFR2* fusions or rearrangements [95]. The approval is based on data from the phase II single-arm FOENIX-CCA2 trial of patients with unresectable or metastatic *FGFR2*-fusion-or-rearrangement-positive iCCA. ORR was 42% (95% CI: 32–52) with a DOR of 9.7 months (95% CI: 7.6 to 17.0) and disease control rate (DCR) of 83% [94]. Median OS was 21.7 months (95% CI: 14.5–not reached) and median PFS was 9.0 months (95% CI: 6.9–13.1) [94]. Even though we cannot compare across trials, ORR appears to be slightly better than pemigatinib (35.5%) and infigratinib (23.1%). In total, 78% of patients had *FGFR* fusions and 22% had *FGFR* rearrangements. *FGFR2-BICC1* followed by *FGFR2-KIAA1217* and *FGFR2-WAC* fusions were the most frequently observed *FGFR2* fusions with 46 unique *FGFR* fusion partners observed in the study population. Adverse events were similar to other FGFR inhibitors [94]. Dose discontinuations were observed in 2% of patients with dose reductions in 54% of patients [94]. Currently, futibatinib is being compared to cisplatin and gemcitabine as a first-line treatment in the phase III trial of advanced CCA harboring FGFR2 gene rearrangements in the FOENIX-CCA3 trial [96]. Table 1 summarizes current FDA approved FGFR inhibitors and trials leading to their approvals. 

## 5. Other FGFR Inhibitors

### 5.1. Selective FGFR Inhibitors

Several other FGFR specific inhibitors have been developed and investigated in numerous trials with variable efficacies and toxicities but none of them have FDA approval yet.

*Rogaratinib* (*BAY1163877*) is an oral FGFR1-4 inhibitor that has been shown to have a favorable toxicity profile in phase I trials of solid tumors overexpressing *FGFR1-3* alterations including HNSCC, NSCLC, and urothelial carcinoma [97]. It was then evaluated in a phase II/III randomized FORT-1 trial compared to chemotherapy (docetaxel, paclitaxel, or vinflunine) in patients with locally advanced or metastatic FGFR1-3 mRNA-overexpressing urothelial carcinoma previously treated with platinum-based therapy [98]. Primary endpoint OS was 8.3 months in the rogaratinib group vs. 9.8 months in the chemotherapy group (HR: 1.11, 95% CI: 0.71–1.72, *p* = 0.67) with ORR of 20.7% in the rogaratinib group vs. 19.3% in the chemotherapy group [98]. It is currently being evaluated in phase I/II trials in urothelial carcinoma and sarcomas/GISTs (NCT03473756, NCT04595747).

*Derazantinib* (*ARQ-087*) is an oral selective FGFR1-3 inhibitor, which has been evaluated in several phase I/II trials, including advanced/inoperable CCA with *FGFR2* gene fusion with ORR of 20.7% and DCR of 82.9% [99]. In a phase II FIDES-01 trial, derazantinib was evaluated in previously treated patients with iCCA and *FGFR2* mutations or amplifications [100]. An interim analysis showed an ORR of 8.7% with stable disease of 65.2% and DCR of 73.9% with higher responses seen in *FGFR2* fusion with ORR of 21.4% and DCR of 75.7% [100]. Derazantinib was also investigated in another phase Ib/II FIDES-02 trial as a single agent in previously treated patients with metastatic urothelial cancer harboring *FGFR1-3* alterations, but derazantinib monotherapy did not meet its primary endpoint with an ORR rate of 8.2% (95% CI: 2.2–19.6) based on 4 PR (all with *FGFR3 S249C* mutation/*FGFR3-TACC3* fusion), and median DOR of 6.9 months [101]. The FEDES-03 trial of derazantinib vs. paclitaxel and ramucirumab or atezolizumab in human epidermal growth factor receptor (HER2)-negative gastric adenocarcinoma with *FGFR2* alterations was terminated due to administrative issues [102]. It is currently being evaluated in combination with atezolizumab in metastatic iCCA and other advanced tumors with FGFR2 alterations (NCT05174650).

*Lirafugratinib* (*RLY4008*) is an oral highly selective irreversible FGFR2 inhibitor with activity across *FGFR2* alterations and resistance mutations [103]. It has been shown to induce responses without clinically significant off-isoform toxicities (hyperphosphatemia, diarrhea), making it a potential FGFR-targeted therapy [103]. It is currently being evaluated in phase I/II trials of patients with unresectable or metastatic CCA and solid tumors harboring *FGFR2* gene fusion, mutation, or amplification in the REFOCUS trial (NCT04526106). It has so far shown high and durable responses in patients with *FGFR2* fusion or rearrangement-positive patients with CCA in three case studies of patients from the REFOCUS trial [103].

*Zoligratinib* (*Debio1347*) is an oral highly selective FGFR1-3 inhibitor that has shown anti-tumor efficacy (ORR of 12% with 20% stable disease) and tolerability in a phase I dose-escalation trial in patients with advanced solid tumors harboring *FGFR* alterations [104].

*Fisogatinib* (*BLU554*) is a highly potent and selective oral FGFR4 inhibitor that covalently binds a cysteine residue found in the FGFR4 [105]. Fisogatinib was evaluated in a phase Ib/II trial in patients with locally advanced or metastatic hepatocellular carcinoma (HCC) [105]. It showed an ORR of 17% in patients with *FGF19* with a median DOR of 5.3 months and median PFS of 3.3 months at a maximal tolerable dose of 600 mg daily [105].

*Aflofanib* (*RPT835*) is a novel selective allosteric FGFR2 inhibitor, which has been evaluated in breast, ovarian, and gastric cancers [106,107]. In a phase Ib trial of previously treated patients (at least one prior line) with metastatic gastric adenocarcinoma, it showed acceptable tolerability and some clinical efficacy with an ORR rate of 9.5% and DCR of 71.4% [106].

*Roblitinib* (*FGF401*) is a selective reversible covalent inhibitor of FGFR4. Roblitinib in combination with spartalizumab showed ORR of 16% in phase I/II trials of patients with HCC or FGFR4/KLB-expressing tumors [108]. The most frequent toxicities were diarrhea and increased aspartate aminotransferase and alanine aminotransferase levels [108].

*Fexagratinib* (*AZD4547*) is an oral selective FGFR1-3 inhibitor, which has been evaluated in several phase I/II studies. In the phase II trial of previously treated patients with FGFR pathway-activated stage IV squamous lung cancer (Lung-MAP substudy), fexagratinib has shown to have acceptable tolerability but has poor efficacy and minimal DOR with only one PR each in a patient with an *FGFR3 S249C* mutation and a patient with *FGFR1* amplification [109]. In another phase II basket trial (NCI-MATCH) of tumors including breast, urothelial, and cervical cancers harboring *FGFR1-3* aberrations, fexagratinib did not meet its primary endpoint and demonstrated ORR of only 8% (90% CI: 3–18%) with responses observed only in patients with *FGFR1-3* point mutations or fusions. Stable disease was seen in 37.5% of patients (90% CI: 25.8% to 50.4%) [110]. Fexagratinib in combination with anastrozole or letrozole showed an ORR of 10% in a single-arm phase II study of patients with ER-positive metastatic breast cancer who progressed on prior hormone therapy, meeting its primary endpoint. However, 20% of patients had reversible and 2% had irreversible asymptomatic retinal pigment epithelial detachments [111].

### 5.2. Multitarget Tyrosine Kinase Inhibitors including FGFR

Some of the multi-TKIs also target FGFR at variable degrees. Please see Table 2 for examples of multi-TKI, which have been investigated and utilized in various types of cancers.

### 5.3. FGFR Ligand Trap

FGFR ligand traps facilitate the binding and trapping of an FGF ligand with the decoy receptors that express the extracellular kinase domain only, thus preventing FGF ligand binding to an FGFR receptor and downstream activation of the FGF pathway [6]. FP1039 (GSK3052230) is a soluble FGFR1 decoy receptor, formed by the fusion of the FGFR1 extra-cellular domain and the human immunoglobulin G, IgG1 Fc fragment. It is effective against *FGFR2*-mutated endometrial and lung cancer cells as well as mesothelioma cell lines with *FGFR1* amplification in pre-clinical studies [117,118,119]. The phase 1b trial evaluated FP1039 in combination with pemetrexed and cisplatin in 36 patients with treatment-naïve, unresectable malignant pleural mesothelioma at doses of 10, 15, and 20 mg/kg. It demonstrated an overall ORR of 39% (95% CI: 23.1–56.5), and DCR of 86%, while ORR was highest at 44% (95% CI: 24.4–65.1) in patients treated with 15 mg/kg of FP1039 [8].

### 5.4. FGFR Monoclonal Antibody

FGFR monoclonal antibodies are developed to target the extracellular domain of FGFR and interfere with ligand binding and receptor dimerization.

*Vofatamab* (*B-701, MFGR1877S*) is a fully human IgG1 monoclonal antibody against FGFR3 [120]. Evaluation of vofatamab in phase I trials of patients with R/R multiple myeloma and t(4;14) translocation causing FGFR3 overexpression and advanced solid tumors showed a tolerable safety profile but without an impressive response [121,122]. Stable disease was the best response achieved in 6/14 patients with myeloma and 9/26 patients with advanced solid tumors including 5 patients with urothelial carcinoma, 2 patients with adenoid cystic carcinoma, and 2 patients with carcinoid tumors [121,122]. In the phase Ib/II FIERCE-21 trial, vofatamab was tolerated either as monotherapy or in combination with docetaxel in previously treated patients with metastatic urothelial cancer (at least one prior line with chemotherapy including taxanes) with *FGFR3* alterations (mutations/fusions). However, it showed minimal single-agent activity with ORR of 11% in heavily pretreated patients [120]. The most common adverse events were decreased appetite, diarrhea, asthenia, hypotension, and increased creatinine [120]. The phase Ib/II FIERCE-22 trial of vofatamab in combination with pembrolizumab in previously treated metastatic urothelial cancers with *FGFR* alterations showed an ORR of 30% (more than the reported response of 20% to immune checkpoint inhibitors (ICIs)). It may be because FGFR inhibition could enhance antigen expression and antigen T-cell clonality, making ICI more effective [123]. Another novel agent, [225Ac]-FPI-1966, is a targeted alpha therapeutic composed of vofatamab, a bifunctional chelate, and actinium-225, an alpha particle emitting radionuclide, and it is currently being investigated in a phase 1 trial of advanced solid tumors (NCT05363605) with *FGFR3* alterations.

*Bemarituzumab* (*FPA144*) is a first-in-class recombinant FGFR2b targeting the humanized IgG1 kappa monoclonal antibody [124]. It binds to the third immunoglobulin region of the FGFR2b receptor, blocks the activation of FGFR2b and downstream FRS2 (fibroblast growth factor receptor substrate 2) phosphorylation, as well as enhances the antibody-dependent cellular toxicity against tumor cells that express FGFR2b [124]. In the phase II FIGHT trial of bemarituzumab in combination with mFOLFOX6 (modified 5-fluorouracil, leucovorin, and oxaliplatin) as a first line in *FGFR2b*-positive advanced gastric/gastroesophageal (GE) junction adenocarcinomas, patients with FGFR2b overexpression irrespective of circulating DNA gene amplification showed improved OS. The bemarituzumab plus chemotherapy arm had better ORR (53% vs. 40%) and better OS (19.2 months vs. 13.5 months) compared to the chemotherapy-alone arm [125]. OS was even higher at 25.4 months in a subset of patients with ≥10% FGFR2b overexpression by immunohistochemistry (2/3+) compared to 11 months in the chemotherapy-alone group [125]. The analysis of PFS was reported in 2022, with a median PFS of 9.5 months in the bemarituzumab and chemotherapy group vs. 7.4 months in the chemotherapy-alone group, which met its primary endpoint but was not statistically significant [124]. However, it showed promising clinical efficacy [124]. The most common grade 3 or worse adverse effects were neutropenia, cornea disorder, and stomatitis. However, hyperphosphatemia or retinal detachments were not seen as much as in other FGFR-TKIs [124]. Currently, bemarituzumab in combination with mFOLFOX is being compared to mFOLFOX alone in untreated, unresectable, locally advanced, or metastatic FGFR2b overexpressed gastric or GE junction adenocarcinoma in the phase III FORTITUDE-101 trial (NCT05052801). In the phase III FORTITUTE-102 trial (NCT05111626), bemarituzumab with mFOLFOX and nivolumab is being compared to mFOLFOX and nivolumab in previously untreated advanced gastric and GE junction cancer with FGFR2b overexpression [126].

### 5.5. Antibody Drug Conjugates

*LY3076226* is an ADC composed of a human IgG1 monoclonal antibody against FGFR3 linked to the cytotoxic payload, maytansine derivative ravtansine (DM4) [118]. LY3076226 was evaluated in the first-in-human phase I trial of patients with advanced or metastatic cancer. It was well tolerated with no dose-limiting toxicities and mostly grade 1 or 2 adverse effects but lacks efficacy (ORR: 0%) [118].

*Aprutumab ixadotin* (*BAY1187982*) is another ADC that consists of a fully human anti-FGFR2 monoclonal antibody attached to an auristatin-like cytotoxic payload [127]. Even though it showed efficacy with inhibition or regression of gastric and breast cancer xenograft models, leading to the phase I trial, it was terminated early due to poor tolerability with dose-limiting toxicities including proteinuria, nephropathy, thrombocytopenia, and epithelial microcytosis [127]. Therefore, there is a need for improved clinical models to predict the effects of investigational ADCs and their metabolites in humans during preclinical development.

Another ADC composed of a tetravalent anti-FGFR1 antibody (T-Fc) linked to a cytotoxic drug, monomethyl auristatin E (MMAE, a tubulin inhibitor), linked via a lysosomally cleavable dipeptide, valine–citrulline (vc), has been recently under development [128]. T-Fc mediates the clustering of FGFR1, leading to the uptake of FGFR1-T-Fc complexes through the induction of clathrin-independent endocytic routes, and they have been shown to have effective drug delivery and internalization by FGFR1-producing cells, leading to the cells’ death [128]. There is potential for the development of ADC with highly effective internalization into FGFR-producing cells and effective killing of cancer cells with tolerable toxicity. Figure 2 summarizes various types of FGFR inhibitors.

## 6. Resistance Mechanisms to FGF/FGFR Pathway Inhibitors

The underlying mechanisms that potentiate FGF/FGFR signaling pathway-related resistance can be associated with various factors. These resistance mechanisms include overexpression of ligands and receptors, downregulation of negative regulators, epithelial–mesenchymal transformation, nuclear translocation, and activation of downstream signaling [129]. Primary resistance occurs due to the initial lack of response to treatment while secondary resistance occurs after the initial response to treatment. *FGFR* gatekeeper mutations are one of the first mechanisms of secondary resistance to FGFR inhibitors. Gene amplifications lead to FGFR overexpression and receptor accumulation, causing continuous downstream signaling pathway activation, including ligand-dependent and -independent pathways [130]. Increased FGF expression from the tumor cell or microenvironment can also overstimulate FGFR and downstream signaling. Furthermore, FGFR inhibition can propagate negative feedback mechanisms that lead to downstream inhibitor resistance. Inhibition of other RTK signaling independent of FGFR signaling could bypass FGFR inhibition. FGFR inhibition induced the activation of Erb-B2 receptor tyrosine kinase 3 (ERBB2) and to a lesser extent EGFR. Consequently, PI3K-AKT signaling is activated, thereby possibly blunting the effects of FGFR inhibitors [131]. Overactivation of the PI3K-AKT pathway via deletion of PTEN is also known to be associated with acquired resistance to FGFR inhibitors [132].

**Figure 3:**
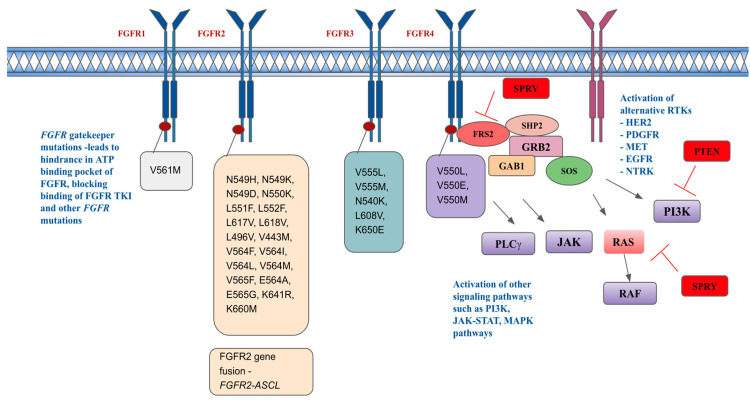
Resistance mechanisms and acquired FGFR-resistant mutations.

Another resistance mechanism is thought to be that FGF/FGFR signaling may contribute to epithelial–mesenchymal transition (EMT), which is the morphological changes defined by cells becoming more spindle-shaped, leading to more potential resistance. Grygielewicz and colleagues observed these morphological changes in the gastric cancer cell line SNU-16 (*FGFR2* amplification), following chronic exposure to medications like infigratinib [133]. Another mechanism for resistance is the nuclear translocation of *FGF* or *FGFR* causing gene fusions. A novel *FGFR2-ACSL5* fusion was identified by Kim et al. in a patient with metastatic gastric cancer and *FGFR2* amplification via RNA sequencing [131,134]. This patient demonstrated strong sensitivity to the study drug during the initial phase of FGFR inhibitor treatment and no *FGFR2-ACSL5* fusion was found in vivo. However, drug resistance was detected along with the *FGFR2-ACSL5* fusion gene after a long exposure to the study drug. Gene fusions can also indirectly lead to FGFR inhibitor resistance. With *JHDM1D-BRAF* fusion, constructive dimerization of the fusion protein is enhanced and is accompanied by the activation of the downstream MAPK pathway. This led to the disappearance of FGFR2 phosphorylation, and a decrease in FGFR2 expression [73]. Further mechanisms of resistance include the downregulation of negative feedback proteins, such as SPRY, which leads to continuous activation of FGF/FGFR signaling. Figure 3 describes resistance mechanisms to FGFR inhibitors.

Figure 3 depicts resistance mechanisms in FGFR pathways, including acquired FGFR kinase mutations, activation of other signal pathways, and activation of alternative RTKs [135,136,137]. HER2 is human epidermal growth receptor 2, PDGFR is platelet-derived growth factor receptor, MET is hepatocyte growth factor receptor, EGFR is epidermal growth factor receptor, NTRK is neurotrophic tyrosine receptor kinase.

## 7. Future Therapeutic Combinations with FGF/FGFR Inhibitors

Given that a single-agent FGFR inhibition treatment could cause intrinsic and acquired resistance, the combination of FGFR inhibitors with chemotherapy, immunotherapy, and targeted therapy combination could be considered for both a synergistic effect and reduction in drug resistance development [4]. FGFR inhibitors can enhance tumor sensitivity to chemotherapy drugs including irinotecan, paclitaxel, 5-fluorouracil, and etoposide in human oncogenic cells with aberrant FGFR activation in in vitro studies [138]. Thus, a combination of chemotherapy with FGFR inhibitors is a consideration.

Dovitinib was investigated in combination with fulvestrant in postmenopausal women with hormone receptor (HR)-positive, HER2-negative, *FGFR* (*FGFR1, FGFR2*, or *FGF3*)-amplified breast cancer who progressed on endocrine therapy in a phase II trial [139]. This trial showed that its combination with fulvestrant in patients with *FGFR* amplification had significantly better PFS of 10.9 months in the dovitinib arm vs. 5.5 months in the placebo arm [139]. Prolonged estrogen deprivation in breast cancer cells can lead to upregulation of FGFR1 together with FGF3, FGF4, and FGF19 due to co-amplification of the *FGFR* gene and genes located in the 11q13 region [140]. More *FGFR1* amplification and treatment-induced FGFR1 overexpression were found in patients with letrozole-resistant HR-positive breast cancer [140].

On the other hand, a combination of FGFR inhibitors with mTOR inhibitors can be considered in some patients who progressed from FGFR inhibitors due to mutations in the FGFR kinase domain. There seems to be PI3K pathway upregulation in cells harboring the FGFR2 p.E565A mutation. Therefore, combination therapy of FGFR and mTOR inhibitors may be considered to overcome resistance to FGFR inhibition [74]. The synergic activity of both FGFR and mTOR inhibitors has been demonstrated in cells harboring HNSCC, lung cancer, and HCC [141,142]. An analysis of circulating tumor DNA (ctDNA) from patients enrolled in the MONALEESA-2 trial of ribociclib demonstrated that patients with *FGFR1* amplification exhibited a shorter PFS compared to patients with wild-type *FGFR1*, thus suggesting *FGFR1* as a mechanism of drug resistance to CDK 4/6 inhibitors and hormone therapy [42]. FGFR multi-TKI lucitanib has shown promising activity in overcoming that resistance; thus, there is potential for the combination use of CDK4/6 inhibitors with FGFR inhibitors in breast cancers with FGFR pathway alterations [42]. In addition, a combination of erdafitinib, palbociclib, and fulvestrant has resulted in complete responses in *FGFR1*-amplified, HR-positive patient-derived xenografts [42,143]. Further investigations in this combination led to an ongoing phase Ib clinical trial evaluating a combination treatment of fulvestrant, palbociclib, and erdafitinib in patients with endocrine-resistant HR-positive, HER2-negative metastatic breast cancer and *FGFR* amplification (NCT03238196). Futibatinib is also currently being evaluated either alone or in combination with fulvestrant in patients with metastatic breast cancer and FGFR alterations in NCT04024436.

Immunotherapy such as anti-PDL1 therapy has been shown to have more effect in *FGFR* wild-type tumors in bladder cancer cell lines while less effects from immunotherapy were observed in FGFR4-overexpressed gastric cancer cells [144,145]. Patients with FGFR1-overexpressed melanoma were also found to have less response to pembrolizumab while *FGFR*-altered HCC tends to have progressive disease after immunotherapy [146,147,148]. Patients with *FGFR* alterations seemed to be less responsive to immunotherapy but 59% of those patients with prior immunotherapy failure had responded to erdafitinib in the BLC2001 trial of erdafitinib [88]. The non-T-cell inflamed subtype of urothelial carcinoma with *FGFR3* mutations was found to have low to absent CD8+ T-cells in the TME, resulting in resistance to ICI monotherapy [149]. All those findings have led to the utilization of immunotherapy and FGFR inhibitor combination in cancer treatments. However, this area of research is still controversial given that some studies did not show the effects of *FGFR* alterations on responsiveness to the immunotherapy [150]. Regardless, this is a novel and exciting field that needs further investigation.

A combination of erdafitinib and anti-PD1 (programmed cell death protein 1) therapy in an indigenous FGFR2K660N/p53mutant lung cancer mouse model demonstrated that combination treatment led to significant tumor regression and improved survival when compared to either treatment alone [151]. The enhanced antitumor activity was supposed to be due to decreased expression of PD1, increased T-cell infiltration, T-cell clone proliferation, and alteration of the TME by immunological changes mediated by erdafitinib [150]. A similar finding of TME regulation and enhancement of T-cells’ cytotoxic effects was seen in lenvatinib combination with anti-PD1 therapy in HCC cells [152]. Pembrolizumab had been evaluated in combination with pemigatinib in patients with advanced cancer and *FGFR* alterations as well as in combination with vofatamab in metastatic urothelial cancers [123,153]. Both combinations have shown good tolerability and there are currently several phase I/II trials investigating the combination of FGFR inhibitors and immunotherapy. Investigation of derazantinib in combination with atezolizumab in patients with *FGFR*-altered urothelial cancers in the phase I/II FIDES-02 trial (NCT04045613) was recently completed and results are pending [154]. Bemarituzumab in combination with chemotherapy and nivolumab (NCT05111626) is also being evaluated in advanced gastric or GE junction cancers with FGFR2b overexpression as mentioned above.

## 8. Mechanism and Management of the Most Relevant Toxicity

The toxicity profile of non-selective FGFR inhibitors is similar to that of VEGFR TKIs, which include fatigue, anorexia, pyrexia, diarrhea, arthralgia, liver toxicity, hypertension, proteinuria, thrombotic microangiopathy, and hypothyroidism. Selective FGFR TKIs can cause hyperphosphatemia, nail disorder with onycholysis, alopecia, mucosal dryness, mucositis, dry eye, conjunctivitis, keratitis, asymptomatic retinal pigment epithelial detachment, osteoarticular pains, myalgias, and muscle cramps [155].

Hyperphosphatemia is a very common adverse effect of FGFR inhibitors because the FGFR1 signaling pathway is a fundamental mechanism to limit the phosphate reabsorption in the proximal renal tubule by inhibiting the phosphate co-transporters [156]. In addition, FGF23 blocks the conversion from 25-hydroxyvitamin D to 1,25-dihydroxyvitamin D in normal physiology. Therefore, FGFR inhibitors increase the 1,25-dihydroxyvitamin D and increase the phosphate absorption from the intestine [156]. The serum phosphate level needs to be monitored closely. Phosphate binding therapy and diet modification are routinely used to lower the phosphate level. Grade 1 toxicity is defined as a sharp rise of 25% above the baseline level (3.5–5.5 mg/dL). Grade 2 is defined as 5.5–6.9 mg/dL while grade 3 is 7–9.9 mg/dL and grade 4 is >10 mg/dL. FGFR inhibitor treatment needs to be held when patients have adverse effects of grade 3 or 4. Acetazolamide can be used in severe cases [156].

Diarrhea is another common adverse effect of FGFR inhibitors because the FGFR pathway regulates bile acid production via a negative feedback mechanism. Bile acids are shown to stimulate the FGF19/FGFR4/ERK1/2 signaling pathway, which in turn causes a negative feedback mechanism. FGFR inhibitors affect this process, resulting in increased production of bile acid, and increased gastrointestinal motility and secretion [156]. Supportive measures with intravenous or oral fluid replacement, probiotics, anti-diarrheal medications, and electrolyte corrections are recommended [156].

Fatigue is also commonly reported but its mechanism is not well understood. Skin, nail, and mucosal changes are also associated with FGFR inhibitors. Topical steroid cream, moisturizers, oral hygiene, and non-alcohol-containing mouthwash are recommended [77,157]. Ocular toxicities such as retinal detachment, central serous retinopathy, dry eyes, and cataracts are significant adverse effects of FGFR inhibitors to be monitored [77,88]. A baseline pretreatment comprehensive eye exam is recommended, and symptomatic patients need to be monitored closely. FGFR inhibitors should be discontinued if ocular toxicities are grade 3 or higher. Ocular toxicities are reversible upon discontinuation of treatment and thus close monitoring and timely discontinuation of an FGFR inhibitor are important [158]. FGFR inhibitors can be restarted at a lower dose if grade 1-2 ocular toxicities resolve [156].

## 9. Ongoing Investigations

The landscape of FGFR inhibitors has evolved over the past decade with the approval of FGFR inhibitors in recent years and there is more to come with several ongoing clinical trials as of this writing (refer to Table 3 for a summary of selected current ongoing clinical trials).

## 10. Conclusions

FGFR inhibitors have shown efficacy in tumors with FGFR alterations in several clinical trials including patients with hematological conditions, iCCA, lung cancers, and urothelial carcinomas. Challenges faced so far are difficulties in patient selection, molecular detection of *FGFR* alterations, acquired resistance of FGFR inhibitors, and management of toxicities [7]. For instance, for gastric cancer, tumor heterogeneity is a challenge, affecting the accuracy of *FGFR2* amplification or overexpression and clinical applications for therapeutic targeting. As tissue heterogeneity poses challenges for molecular diagnostic testing, ctDNA is currently under investigation as a potential modality with comparable rates of detection to tissue-based methods as seen in the GOZILA study [159].

Despite the efficacy of FGFR inhibitors, acquired resistance occurs due to the devolvement of secondary mutations in the FGFR kinase domain, making it resistant to infigratinib or pemigatinib. Third-generation irreversible FGFR inhibitors such as futibatinib can overcome those mutations but a cysteine mutation could still occur [7]. Acquired mutations in the kinase domain could be avoided via the development of FGFR kinase allosteric or specific fusion partner inhibitors, such as TACC3-targeting inhibitors (BO-264), which can inhibit the growth of cells harboring *FGFR3-TACC3* fusions [160]. In addition, FGFR and fusion partners can be degraded by FGFR-targeting proteolysis targeting chimera (PROTACs), thus avoiding inhibitor-induced acquired mutation [7].

On the other hand, we could consider the combination of FGFR inhibitors with mTOR inhibitors in some patients who progressed on FGFR inhibitors due to mutations in the FGFR kinase domain. As discussed earlier, there is upregulation of the PI3K/AKT/mTOR signaling pathway in cells harboring the *FGFR2* mutation. Thus, combination therapy of FGFR and mTOR inhibitors may overcome resistance to FGFR inhibition [74]. Combination of FGFR inhibitors with EGFR or MAPK pathway inhibitors can also be considered given that FGFR inhibitor AZD4547 has synergic activity with EGFR inhibitor cetuximab in gastric cancer cells with *FGFR2* amplifications [161]. Given acquired mutations and resistance, there is a need for a more comprehensive understanding of mutations that develop in response to FGFR inhibition and the sensitivity of current inhibitors to develop novel inhibitors.

In addition, future investigations into combination treatments with immunotherapy and optimal sequencing of immunotherapy and FGFR inhibitors in *FGFR*-altered cancers are also needed given that ORR of erdafitinib was higher in patients with urothelial cancers previously exposed to ICI. FGFR monoclonal antibody bemarituzumab is currently under a few phase III clinical trials to be used as first-line treatment in gastric or GE junction cancers. However, further advances in ligand traps and ADCs are not well developed yet.

FGFR inhibitors have been shown to have efficacy across various types of cancers with *FGFR* alterations, and currently several approved and novel FGFR inhibitors are being investigated in various clinical trials, either alone or in combinations with other therapies. Despite their limitations due to limited responses, acquired resistances, and intolerable toxicities, they have the potential to treat various cancers with *FGFR* alterations and overcome resistance to other anti-cancer treatments. Thus, further investigations are needed to develop FGFR inhibitors that have better and more durable responses with more tolerable toxicities and the ability to overcome acquired resistance. Further developments of FGFR inhibitors will pave the way for personalized medicine in which individualized treatments are given based on molecular profiling of tumors.

## Figures and Tables

**Table 1:** Current FDA-approved FGFR inhibitors with supporting trials for FDA approval.

Drug	Trial	Phase	Study Population, Number (n)	ORR (%)	mDOR Months	mOS Months	mPFS Months	FDA Approval	Adverse Effects
Pemigatinib FGFR1-3 inhibitor	Abou-Alfa et al. FIGHT-202 trial [77,78]	II	Locally advanced, unresectable, or metastatic CCA with *FGFR2* gene fusion or rearrangements, progressed on at least one prior line of therapy. n = 146	35.5	9.1	17.5	-	17 April 2020 as second line	Hyperphosphatemia, alopecia, dysgeusia, diarrhea, fatigue, stomatitis, dry mouth, arthralgia, hyponatremia, abdominal pain, fatigue, pyrexia, cholangitis, and pleural effusion
	Gotlib et al. FIGHT-203 trial [81]	II	MLNs with *FGFR1* rearrangement regardless of prior lines of treatment. n = 34	64.7 (CR)	Not reached	-	-	26 August 2022 as second line	Hyperphosphatemia, alopecia, diarrhea, stomatitis, and anemia
Infigratinib FGFR1-3 inhibitor	Javle et al. CBGJ398X2204 trial [85]	II	Locally advanced, or metastatic CCA with *FGFR2* fusions or rearrangements, progressed on at least one prior line. n = 108	23.1	5	-	7.3	28 May 2021 as second line	Hyperphosphatemia, eye disorders, hyponatremia, stomatitis, and fatigue
Erdafitinib FGFR1-4 inhibitors	Siefker-Radtke et al. BLC2001 trial [89]	II	Locally advanced, unresectable, or metastatic urothelial cancers with *FGFR* alterations, progressed on at least prior line or within 12 months after neoadjuvant or adjuvant chemotherapy. n = 99	40	5.6	13.8	5.5	12 April 2019 as second line	Hyperphosphatemia, stomatitis, diarrhea, and dry mouth, hyponatremia, and asthenia
Futibatinib FGFR1-4 inhibitor	Goyal et al. FOENIX-CCA2 trial [94]	II	Locally advanced, unresectable, or metastatic iCCA with *FGFR2* fusions or rearrangements that progressed on at least one prior line. n = 109	42	9.7	21.7	9.0	30 September 2022 as second line	Hyperphosphatemia, alopecia, dry mouth, diarrhea, dry skin, fatigue, palmer-planter erythrodysesthesia syndrome, increased aspartate aminotransferase level, and stomatitis

**Table 2:** Multi-target tyrosine kinase inhibitors.

Multi-TKIs	Targets	Reference
Dovitinib (TKI258)	FGFR, VEGFR, PDGFR inhibitor	[112]
Lenvatinib (E7080)	VEGFR1-3, FGFR1-4, PDGFR α, RET, KIT	[113]
Lucitanib (E-3810)	FGFR1-2, VEGFR1-3, and PDGFRα-β	[114]
Pazopanib (GW786034)	FGFR1-2, VEGFR1-3, PDGFRα-β, C-kit (stem cell factor receptor)	[115]
Ponatinib (AP24534)	FGFR1-4, VEGFR2, PDGFRα, c-SRC, c-Kit, FLT3, RET	[116]

**Table 3:** Selected current clinical trials.

Agent	NCT	Status	Conditions	Phase
Pemigatinib	NCT03914794	R	Non-muscle invasive bladder cancer (NMIBC) with recurrent low or intermediate risk tumors (as neoadjuvant)	II
Pemigatinib	NCT03011372	A, NR	Previously treated myeloid/lymphoid neoplasms with *FGFR1* rearrangement (FIGHT-203)	II
Pemigatinib vs. gemcitabine + cisplatin	NCT03656536	R	Untreated unresectable or metastatic cholangiocarcinoma with *FGFR2* rearrangement (FIGHT-302)	III
Pemigatinib	NCT05565794	R	Intrahepatic cholangiocarcinoma with *FGFR2* gene mutation, rearrangement, or translocation after curative local therapy	II
Pemigatinib	NCT05267106	R	Previously treated glioblastoma or other primary central nervous system tumors harboring activating *FGFR1-3* alterations (FIGHT-209)	II
Pemigatinib	NCT05253807	A, NR	Relapsed or refractory advanced non-small cell lung cancer with an *FGFR* alteration (FIGHT-210)	II
Pemigatinib	NCT04659616	R	Acute myeloid leukemia after initial induction chemotherapy with adverse or intermediate risk cytogenetics	I
Futibatinib	NCT04189445	A, NR	Previously treated advanced or metastatic solid tumors, gastric or gastroesophageal cancers, myeloid or lymphoid neoplasms with *FGFR1-4* rearrangements	II
Futibatinib +/− fulvestrant	NCT04024436	A, NR	Previously treated metastatic breast cancer with *FGFR1* and *FGFR2* amplification	II
Futibatinib vs. gemcitabine + cisplatin	NCT04093362	A, NR	Previously untreated advanced cholangiocarcinoma harboring *FGFR2* gene rearrangements (FOENIX-CCA3)	III
Futibatinib	NCT05727176	R	Previously treated advanced cholangiocarcinoma with *FGFR2* fusion or rearrangement (FOENIX-CCA4)	II
Futibatinib + pembrolizumab	NCT04828486	R	Advanced or metastatic hepatocellular carcinoma with FGF19 expression	II
Futibatinib + pembrolizumab	NCT04601857	R	Advanced or metastatic urothelial carcinoma that are not candidates to receive a platinum-based treatment regimen	II
Futibatinib + pembrolizumab	NCT05036681	R	Previously treated locally advanced or metastatic microsatellite stable endometrial carcinoma	II
Infigratinib	NCT04233567	A, NR	Previously treated advanced or metastatic solid tumors, cholangiocarcinoma, and refractory malignant solid neoplasm with *FGFR* gene mutations	II
Infigratinib	NCT04228042	A, NR	For renal pelvis and upper tract urothelial cancer as neoadjuvant treatment	I/II
Erdafitinib	NCT04917809	R	Recurrent non-invasive bladder cancer with *FGFR3* gene mutation after treatment with instillations of BCG or chemotherapy into the bladder	II
Erdafitinib	NCT04083976	A, NR	Advanced or metastatic solid tumors with *FGFR* alterations (mutations or gene fusions) after at least one prior line of systemic therapy (RAGNAR)	II
Erdafitinib	NCT04754425	R	Castration-resistant prostate cancer after progression on second-generation androgen receptor targeting agents	II
Erdafitinib + fulvestrant + palbociclib	NCT03238196	A, NR	Previously treated HR-positive HER2-negative *FGFR*-amplified metastatic breast cancer	I
Fisogatinib	NCT02508467	A, NR	Hepatocellular carcinoma with FGF19 expression with or without prior tyrosine kinase inhibitors	I
Derazantinib + atezolizumab	NCT05174650	R	Previously treated advanced or metastatic intrahepatic cholangiocarcinoma with *FGFR2* fusions/rearrangements	II
Lirafugratinib (RLY4008)	NCT04526106	R	Previously treated unresectable/metastatic intrahepatic cholangiocarcinoma and other advanced tumors with *FGFR2* alterations (REFOCUS)	I/II
Dovitinib + PARP inhibitor stenoparib (2X-121)	NCT05571969	R	Advanced solid tumors	I
Rogaratinib (BAY1163877) + atezolizumab	NCT03473756	A, NR	Cisplatin-ineligible patients with metastatic or locally advanced urothelial carcinoma and *FGFR1* or *3* alterations (FORT-2)	Ib/II
Rogaratinib	NCT04595747	A, NR	Previously treated advanced or metastatic sarcoma with *FGFR1-4* alterations and in patients with SDH-deficient gastrointestinal stromal tumor (GIST)	II
Fexagratinib AZD4547 + tislelizumab	NCT05775874	R	Locally advanced or metastatic urothelial cancer with *FGFR2/3* alterations in Chinese patient population	II
Bemarituzumab	NCT05325866	R	Refractory or relapsed advanced or metastatic solid tumors with FGFR2b overexpression after at least one prior line (FORTITUDE-301)	I
Bemarituzumab + mFOLFOX6 + nivolumab vs. mFOLFOX6 + nivolumab	NCT05111626	R	Untreated advanced or metastatic gastric and gastroesophageal junction (GEJ) cancer with FGFR2b overexpression (FORTITUDE-102)	III
Bemarituzumab + mFOLFOX vs. mFOLFOX	NCT05052801	R	Previously treated advanced or metastatic gastric or GEJ cancers with FGFR2b overexpression (FORTITUDE-101)	III
Bemarituzumab + anti-cancer therapy	NCT05267470	A, NR	Advanced or metastatic squamous lung cancer with FGFR2b overexpression (FORTITUDE-201)	I

A = Active, NR = Non-recruiting, R = Recruiting. Retrieved from clinicaltrials.gov [102].

## Data Availability

Not applicable.
